# Protocol for development and validation of postpartum cardiovascular disease (CVD) risk prediction model incorporating reproductive and pregnancy-related candidate predictors

**DOI:** 10.1186/s41512-022-00137-7

**Published:** 2022-12-19

**Authors:** Steven Wambua, Francesca Crowe, Shakila Thangaratinam, Dermot O’Reilly, Colin McCowan, Sinead Brophy, Christopher Yau, Krishnarajah Nirantharakumar, Richard Riley, on behalf of the MuM-PreDiCT Group

**Affiliations:** 1 Institute of Applied Health Research, College of Medical and Dental Sciences, University of Birmingham, Edgbaston, Birmingham, UK; 2 WHO Collaborating Centre for Global Women’s Health, Institute of Metabolism and Systems Research, University of Birmingham, Birmingham, UK; 3 Department of Obstetrics and Gynaecology, Birmingham Women’s and Children’s NHS Foundation Trust, Birmingham, UK; 4 Centre for Public Health, Queen’s University Belfast, Belfast, UK; 5 School of Medicine, University of St Andrews, St Andrews, UK; 6 Data Science, Medical School, Swansea University, Swansea, UK; 7 Big Data Institute, University of Oxford, Li Ka Shing Centre for Health Information and Discovery, Old Road Campus, Oxford, OX3 7LF UK; 8 Nuffield Department of Women’s & Reproductive Health, University of Oxford, Level 3 Women’s Centre, John Radcliffe Hospital, Oxford, OX3 9DU UK; 9 Health Data Research, London, UK

**Keywords:** Prediction modeling, Cardiovascular disease, Pregnant women, Prognosis, Pregnancy complications

## Abstract

**Background:**

Cardiovascular disease (CVD) is a leading cause of death among women. CVD is associated with reduced quality of life, significant treatment and management costs, and lost productivity. Estimating the risk of CVD would help patients at a higher risk of CVD to initiate preventive measures to reduce risk of disease. The Framingham risk score and the QRISK® score are two risk prediction models used to evaluate future CVD risk in the UK. Although the algorithms perform well in the general population, they do not take into account pregnancy complications, which are well known risk factors for CVD in women and have been highlighted in a recent umbrella review.

We plan to develop a robust CVD risk prediction model to assess the additional value of pregnancy risk factors in risk prediction of CVD in women postpartum.

**Methods:**

Using candidate predictors from QRISK®-3, the umbrella review identified from literature and from discussions with clinical experts and patient research partners, we will use time-to-event Cox proportional hazards models to develop and validate a 10-year risk prediction model for CVD postpartum using Clinical Practice Research Datalink (CPRD) primary care database for development and internal validation of the algorithm and the Secure Anonymised Information Linkage (SAIL) databank for external validation. We will then assess the value of additional candidate predictors to the QRISK®-3 in our internal and external validations.

**Discussion:**

The developed risk prediction model will incorporate pregnancy-related factors which have been shown to be associated with future risk of CVD but have not been taken into account in current risk prediction models. Our study will therefore highlight the importance of incorporating pregnancy-related risk factors into risk prediction modeling for CVD postpartum.

**Supplementary Information:**

The online version contains supplementary material available at 10.1186/s41512-022-00137-7.

## Introduction

CVD is a leading cause of morbidity and mortality globally in both men and women [1, 2]. Estimating the risk of the condition would help patients at a higher risk of CVD to access treatments to reduce the risk of developing CVD. There are several risk prediction models used routinely in primary care to predict CVD risk in the general population. These include the Framingham risk score model and the QRISK® score [3, 4]. However, studies have shown that they tend to underestimate the risk of CVD in young women [5, 6]. While the most recent QRISK® calculator includes several comorbidities (for example diabetes mellitus) and one male-related risk factor (erectile dysfunction), there are no female-specific candidate predictors included in the CVD risk prediction models [5].

During pregnancy, women experience cardiovascular physiological changes such as an increase in cardiac output. A small proportion of pregnant women develop pregnancy-induced hypertension and preeclampsia [7], and a woman’s response to such changes could be linked to future cardiovascular health [8]. Several studies have identified a link between certain pregnancy complications (e.g., gestational hypertension, preeclampsia, placental abruption, preterm birth, gestational diabetes mellitus, and stillbirth) and reproductive health factors (e.g., early age at menarche and polycystic ovary syndrome) with risk of CVD [9–11]. More recently, the postpartum period has been identified as a possible window of opportunity to initiate cardiovascular disease preventative measures in women [12, 13]. However, there is lack of guidelines on risk factor management in this population.

There have been recent efforts to quantify the predictive value of pregnancy-related candidate predictors to established CVD risk prediction models [5, 14–16]. A study by Markovitz et al. [14] showed that adding pregnancy complications history to the NORRISK 2 risk model improved the c-index by 0.004, while another study by Marzieh et al. [15] established that the Framingham risk score was enhanced (c-statistic of 0.0053) after adding these factors. The National Institute for Health and Care Excellence (NICE) recommends using QRISK® assessment tool to calculate a person’s 10-year risk of CVD in the UK, but there have been no attempts to evaluate the added value of pregnancy factors in the development of the risk prediction model in women.

We plan to develop a robust CVD risk prediction model postpartum to assess whether adding reproductive health and pregnancy-related candidate predictors to the QRISK®-3 risk prediction model improves the performance of the individual risk prediction of CVD in women.

### Objectives

The main aim of this study is to update the QRISK®-3 tool to include candidate predictors related to women’s health to help predict the risk of CVD postpartum in women without a history of CVD. This tool will be important to help healthcare professionals in their decision making about the need for targeted care. The specific objectives of the study are as follows:

i. To externally validate the QRISK®-3 score in the postpartum period using a large, representative study population of women from UK primary care
ii. To develop a clinical prediction model for 10-year risk of CVD postpartum (15 months after conception as index date) and internally validate its performance (overall model fit, calibration, and discrimination) using the study population in objective (i)
iii. To externally validate the risk prediction model developed in objective (ii), by examining its performance and clinical utility in a separate large, representative study population of women from UK primary care, both overall and within relevant subgroups

### Research design and methods

#### Data sources

Two databases of anonymized Electronic Health Records will be used for this study. They are as follows:

1. Clinical Practice Research Datalink (CPRD) [17], which has over 19 million patient records in the UK from over 940 participating general practices, with a mean follow-up of 13 years as of February 2021.
  The CPRD pregnancy register is used to capture information from maternity, antenatal, and delivery records to identify pregnancies within CPRD GOLD [18]. According to recent data, the CPRD register captured 5.8 million pregnancies among 2.4 million women in the period January 1987–February 2018 [18]. We will use the register to extract pregnancy data from CPRD.
2. Secure Anonymised Information Linkage (SAIL) [19], which has data from over 4 million patient records in Wales and covers 80% of Welsh general practices [20]. Follow-up is longer than CPRD databases as SAIL tracks patient journeys even when they transfer practice within Wales.
  The National Community Child Health Database will be used to identify pregnancies and will be linked to the Welsh Longitudinal General Practice database (for diagnosis and medications data) and Welsh Demographic Service database (for demographics data) within the SAIL databank. Using these databases, 27,783 pregnant women were identified in SAIL in 2018 in a study conducted within the MuM-PreDiCT consortium [21]. We expect to have more pregnant women within a follow-up period of 10 years.

Both databases contain data from GP practices captured primarily using Vision software. CPRD will be used to develop the risk prediction model and for the internal validation process while the SAIL database will be used for independent external validation of the risk prediction model. We will exclude data on patients from Wales in CPRD to ensure no overlap with patients in the SAIL database.

#### Target population

The target population is women between the ages of 15 and 49 years who have a history of pregnancy and registered with participating GPs between 1 of January 2000 and 31 of December 2021. Women with pre-existing CVD before study entry will be excluded as the risk prediction model is for those who have not been diagnosed with CVD.

Each woman can contribute to the cohort after a minimum registration period with their GP for at least 12 months to ensure sufficient quality data at baseline. The index date will be 15 months after date of conception of the last pregnancy (estimated to be 6 months post-partum). The index date has been chosen to be around 6 months postpartum because this allows for normal physiological changes of pregnancy to resolve and time lag for postpartum information to be recorded in the GP database [22, 23].

Participants will be followed from the index date until the earliest of outcome date, transfer date (CPRD GOLD), last date of data collection, death date, or study end date. Participants will be censored 10 years after the index date.

Flow chart of participants from baseline (6 months postpartum) through completion of the study (study end, 31 December 2021) will be presented in the final report.

#### Study outcome

The outcome will be the first recorded diagnosis of cardiovascular disease (coronary heart disease, stroke, myocardial infarction, or transient ischemic attack). The outcome will be ascertained using Read codes, a clinical terminology system used for record-keeping in general practice in the National Health Service (NHS) [24]. For comparability, Read codes for the outcome of CVD have been obtained from the article on the development and validation of the QRISK®-3 and are presented in Additional file 1.

### Clinical predictor variables

#### Determining candidate predictors for model development

Candidate predictors are features that will be investigated for their potential predictive value towards risk prediction of CVD postpartum. The features will include any information that precedes cardiovascular disease and are available at the start-point (moment of intended prediction) and are linked to an increased risk of CVD. Examples will include pregnancy-related risk factors for example gestational diabetes, pre-eclampsia, and gestational hypertension.

We will use two approaches to identify candidate predictors: (1) clinical and patient expertise and (2) evidence from previous studies [25]. For the clinical and patient expertise approach, candidate predictors will be selected through discussions with clinicians and patient research partners while for the evidence from previous studies approach, and risk factors will be identified through literature review [26]. Potential candidate predictors for CVD postpartum have been chosen based on the umbrella review identified from the literature and clinical significance and through discussions with clinicians and patient research partners. We plan to assess the data quality of the potential candidate predictors chosen, including by evaluating missing data and any outliers, and the timing and method of their measurement. We will then perform variable selection using the least absolute shrinkage and selection operator (LASSO) to determine predictors that will be included in the final model [27, 28].

#### Proposed candidate predictors

Table 1 shows the list of the proposed candidate predictors from QRISK®-3, an umbrella review of reproductive health factors associated with CVD in young women, and from discussions with clinicians and patient research partners [4, 26].

**Table 1:** Proposed candidate predictors

Candidate predictors identified from QRISK-3 relevant to women [4]	Candidate predictors identified from Umbrella Review [26]	Candidate predictors identified from discussions within the research team
**Patient characteristics** • Age • Ethnicity • Deprivation • Systolic blood pressure (SBP) • Body mass index (BMI) • Total cholesterol: high-density lipoprotein cholesterol ratio • Smoking status ***Medical history predictors*** • Family history of coronary heart disease • Diabetes mellitus • Treated hypertension • Rheumatoid arthritis • Atrial fibrillation • Chronic kidney disease (stages 4 or 5) • Expanded definition of chronic kidney disease (to include general practitioner recorded diagnosis of chronic kidney disease stage 3 in addition to stages 4 and 5 as well as major chronic renal disease) • The standard deviation of SBP (variability of repeated SBP measures) • Diagnosis of migraine • Corticosteroid use • Systemic lupus erythematosus • Second generation “atypical” antipsychotic use • Diagnosis of severe mental illness	**Reproductive health factors** • Early age at menarche (<12 years old) • Use of hormonal contraceptive agents • Use of oral contraceptives • Polycystic ovary syndrome • Parity/gravidity **Adverse pregnancy outcomes** • Hypertensive disorders of pregnancy • Gestational diabetes mellitus • Placental abruption • Pregnancy loss • Preterm birth • Low birth weight and small for gestational age	• Idiopathic intracranial hypertension • Postnatal depression • Hypothyroidism • Hyperthyroidism • Endometriosis • Menstrual irregularity • Antiphospholipid syndrome

The list of potential candidate predictors can be expanded to include more potential factors based on new information that emerges from literature or through discussions with clinicians and patients as the project progresses.

### Statistical analysis

*Steps for development and validation of the updated risk prediction model*

i) Externally validate the QRISK®-3 using CPRD-GOLD data
ii) Use the QRISK®-3 model coefficients in (i) above as a single predictor and add additional candidate predictors to develop and internally validate an updated risk prediction model (Model 1).
iii) Develop and internally validate a risk prediction model using all predictors, i.e., QRISK®-3 predictors plus additional candidate predictors (Model 2), allowing for variable selection via the LASSO.
iv) Compare predictive performance measures (calibration and discrimination) of QRISK®-3, Model 1 and Model 2, using internal validation techniques.
v) Externally validate the best risk prediction model (based on predictive performance measures obtained after internal validation) between Model 1 and Model 2, using the SAIL database, and again compare to QRISK®-3
vi) Compare predictive performance measures (calibration, discrimination, and net benefit analysis) in (i) and in (v) above.

#### Development, internal and external validation of models

##### Missing data

Missing data in each candidate predictor will be investigated before analysis. Missing data will be classified based on whether the values are expected to be missing at deployment. Missing data will then be handled using three approaches. Firstly, missing entry for a condition (e.g., diabetes) will be taken to indicate the absence of the comorbidity (no history of diabetes). Secondly, the missing indicator method will be used for variables where we expect informative missingness at deployment. For example, if a biomarker test (e.g., blood pressure measurements, blood cholesterol, HbA1c, etc.) has been carried out, then the perceived need for the test of the biomarker might be informative of the patient’s health [29]. Thirdly, multiple imputations with chained equations will be applied for candidate predictors where we do not expect missing data at deployment. The three approaches will be used to ensure missing data methods match at both the development and deployment stages of the risk prediction model as recommended in recent studies [29].

##### Externally validating QRISK®-3 equation using CPRD-GOLD dataset

The QRISK**®**-3 risk prediction model was developed using the QResearch primary care database [30]. The first step will be to externally validate the QRISK**®**-3 risk prediction model using the CPRD-GOLD dataset and assess its performance for women with a history of pregnancy. This will form a benchmark for risk prediction models incorporating additional candidate predictors.

We will calculate 10-year risk of CVD (predicted risk) for women with a history of pregnancy using the QRISK®-3 algorithm. The observed 10-year risk (observed risk) of CVD will be estimated using the method of Kaplan-Meier. Missing data in each predictor will be handled in a similar way as during the development of the QRISK®-3 [4]. We will examine the predictive performance of QRISK®-3 in the population using calibration (plots, curves, and slope) to see how closely the predicted risk agrees with the observed risk and discrimination (the model’s ability to distinguish between those who develop post-partum CVD and those who do not, summarized as time-dependent C-statistics and Royston’s D statistic). These measures will be obtained overall and for sub-groups of women defined by ethnicity, socio-economic status, and age. We have chosen to validate the risk prediction model within these subgroups for a start because algorithmic biases in the risk prediction models used in healthcare occur in various subgroups defined by ethnicity, socio-economic status, and age [31]. Additionally, previous studies have considered these type of subgroups in the validation of risk prediction models for CVD [4, 32].

##### Primary model development

Using all the candidate predictors identified previously, we will develop our models using a Cox proportional hazards regression (combined with a non-parametric estimate of the baseline survival) following practical approaches for clinical prediction models [33–35]. If competing risks are prevalent, for example due to the risk of dying from causes other than CVD, then this will be accounted for using sub-distribution (Fine Gray) approaches, with the Aalen-Johansen estimator used to obtain the baseline survival [36]. Model parameter coefficients will be pooled across imputations using Rubin Rules to produce the model.

##### Model performance

The main follow-up time-point will be 10 years, but earlier time points (e.g., at 5 years) will also be considered. The choice of the 10-year time point is because NICE guidelines recommend clinicians to offer a statin based on the risk of CVD within 10 years. However, we are also considering shorter-time scale (5 years) as sensitivity analysis and to enable early interventions to reduce the risk of CVD. The initial model will include all candidate predictors (no variable selection). This model will then be compared with a model employing least absolute shrinkage and selection operator (LASSO) for variable selection. Continuous variables will be analyzed on their continuous scale, with non-linear trends modeled using fractional polynomials. Overfitting and optimism are expected to be minimal (due to the large sample size) but will be evaluated using bootstrapping (incorporating all model development steps) and heuristic shrinkage estimates and adjusted for using a uniform shrinkage factor if necessary, to produce the final model.

To evaluate the validity of our data, we will compare the representativeness of our datasets to published CVD populations by summarizing clinical features. Each model’s apparent performance will be evaluated at specific time points of 5 and 10 years post-partum, and, if necessary, recalibration by time will be applied by refitting the models linear predictor to the pseudo-observations at each time-point using a generalized linear model [37], to produce a separate prediction model for each time-point of interest. The ability of the model to correctly classify disease status will be evaluated by calculating the models’ discrimination in terms of time-dependent C-statistics and Royston’s D statistic; the models’ calibration by plotting the observed probability of the outcome against predicted probability (smoothed calibration curves), at particular time-points using the pseudo-value approach [38], alongside summary measures of calibration, and clinical utility across a range of risk thresholds deemed clinically relevant by our user groups.

##### The sample size for development

The CPRD pregnancy register captured 5.8 million pregnancies among 2.4 million women in the period January 1987–February 2018 [18]. We will use the register to extract pregnancy data from CPRD for the period 1 of January 2000 and 31 of December 2021. Once the data are obtained, further assessment will be done to ensure the sample size (and outcome event proportion) available meets the minimum sample size that ensures accurate estimation of regression coefficients and reduces overfitting during model development [39, 40]. Given we anticipate a large sample size, it is highly likely to do this. If not, we will reduce the number of candidate predictors accordingly.

#### External validation of the models

The SAIL database will be used for external validation to evaluate the predictive performance and clinical utility of the newly developed risk prediction model. The performance will be assessed at various time points as a whole and within important subgroups (e.g., age groups, socio-economic status, and ethnicity) using the following predictive performance measures; calibration, discrimination, and clinical utility (using net benefit analysis and decision curves).

The clinical utility of incorporating the risk prediction model into clinical practice will be assessed using decision curves [41]. The net benefit, which is the fraction of true positives gained by making decisions based on risk predictions over a range of possible risk thresholds will be evaluated [42]. We will define the threshold probability as the population risk of CVD postpartum. The net benefit of the risk prediction model will be compared using a decision curve analysis assuming all are at high risk (“treat all [offer a statin to all people who have 10% or greater risk of developing cardiovascular disease within the next 10 years according to NICE guidelines]”) and assume all are at low risk (“treat none”). We will also compare the net benefit of the risk prediction model with current practice guidelines on postpartum CVD.

Missing data will be handled in a similar way as during the development stage of the risk prediction model.

##### The sample size for validation

Using the QRISK®-3 risk prediction model for women, we estimated the distribution of the linear predictor and calculated the minimum sample size needed for external validation of QRISK®-3 using a recommended simulation-based approach for calculating a risk prediction model with a time-to-event outcome [43]. We established that a minimum sample size of about 24,000 patients and 264 CVD events would result in precise estimates of prediction model performance, for example with a calibration slope CI width of 0.3 (i.e., CI width of 0.85-1.15 assuming the true value is 1), with an assumed 20% censoring rate by 10 years. The validation datasets will be evaluated to confirm the number of CVD events postpartum exceeds 264, but this is expected.

#### Statistical software

The computer software programs R version 4.2.1 and Stata (StataCorp. 2021. *Stata Statistical Software: Release 17*. College Station, TX: StataCorp LLC.) will be used for all analyses.

#### Model presentation

The whole study will be reported following Transparent Reporting of a Multivariable Prediction Model for Individual Prognosis or Diagnosis (TRIPOD) guidelines [44].

## Discussion

In the proposed study, we aim to evaluate the added value of reproductive and pregnancy related risk factors, which have been shown to be associated with future risk of CVD but have not been taken into account in current risk algorithm for CVD, QRISK®-3. Our study will therefore highlight the importance of incorporating reproductive and pregnancy-related risk factors into risk prediction modeling post-partum.

Our study will develop and internally validate the risk prediction model developed using a large cohort of primary care data from CPRD. This implies large sample sizes to enable stability of the parameters estimated. We will also use a separate dataset (SAIL) for external validation of the developed risk prediction model and hence quantify the generalizability of the algorithm in the UK population and within relevant subgroups (e.g., age groups, socio-economic status, and ethnicity). This will enable us to detect any algorithmic biases and therefore develop frameworks to reduce them.

We foresee some limitations to this study. While the CPRD database is representative of the UK population, recording rates for primary care record codes may vary significantly between practices. We plan to assess the data quality before analysis. We also expect missing data in some of the candidate predictors, and we have outlined a framework to address this challenge during both the development and validation phases. Finally, although data such as genetics could be potential predictors in the risk prediction modelling of CVD, we will not consider them in our study as there is still little information in primary care datasets.

## Supplementary Information

**Additional file 1.** Read codes to be used to identify patients with CVD from GP records (obtained from QRISK®-3 for comparability of models).

## Data Availability

The datasets that will be used for this study are available from CPRD and SAIL and can be accessed upon reasonable request.
