# Supplementary material for: Protocol for development and validation of postpartum cardiovascular disease (CVD) risk prediction model incorporating reproductive and pregnancy-related candidate predictors
Source: Diagn Progn Res. 2022 Dec 19;6:23. doi: 10.1186/s41512-022-00137-7 (PMC9761974; doi:10.1186/s41512-022-00137-7)
Supplement: Supplementary file 1 — Additional file 1. Read codes to be used to identify patients with CVD from GP records (obtained from QRISK®-3 for comparability of models). [file 41512_2022_137_MOESM1_ESM.docx]

| **List of Appendices** Read codes to be used to identify patients with CVD from GP records (obtained from QRISK®-3 for comparability of models)  \| **Group Name** \| **ReadTerm** \| **ReadDesc** \| \| --- \| --- \| --- \| \| Coronary Heart Disease \| G3 \| Ischaemic heart disease \| \| Coronary Heart Disease \| G3-1 \| Arteriosclerotic heart disease \| \| Coronary Heart Disease \| G3-2 \| Atherosclerotic heart disease \| \| Coronary Heart Disease \| G3-3 \| IHD - Ischaemic heart disease \| \| Coronary Heart Disease \| G30 \| Acute myocardial infarction \| \| Coronary Heart Disease \| G30-1 \| Attack - heart \| \| Coronary Heart Disease \| G30-2 \| Coronary thrombosis \| \| Coronary Heart Disease \| G30-3 \| Cardiac rupture following myocardial infarction (MI) \| \| Coronary Heart Disease \| G30-4 \| Heart attack \| \| Coronary Heart Disease \| G30-5 \| MI - acute myocardial infarction \| \| Coronary Heart Disease \| G30-6 \| Thrombosis - coronary \| \| Coronary Heart Disease \| G30-7 \| Silent myocardial infarction \| \| Coronary Heart Disease \| G30-98 \| Coronary thrombosis \| \| Coronary Heart Disease \| G30-99 \| Myocardial Infarction \| \| Coronary Heart Disease \| G300 \| Acute anterolateral infarction \| \| Coronary Heart Disease \| G301 \| Other specified anterior myocardial infarction \| \| Coronary Heart Disease \| G3010 \| Acute anteroapical infarction \| \| Coronary Heart Disease \| G3011 \| Acute anteroseptal infarction \| \| Coronary Heart Disease \| G301z \| Anterior myocardial infarction NOS \| \| Coronary Heart Disease \| G302 \| Acute inferolateral infarction \| \| Coronary Heart Disease \| G303 \| Acute inferoposterior infarction \| \| Coronary Heart Disease \| G304 \| Posterior myocardial infarction NOS \| \| Coronary Heart Disease \| G305 \| Lateral myocardial infarction NOS \| \| Coronary Heart Disease \| G306 \| True posterior myocardial infarction \| \| Coronary Heart Disease \| G307 \| Acute subendocardial infarction \| \| Coronary Heart Disease \| G3070 \| Acute non-Q wave infarction \| \| Coronary Heart Disease \| G3071 \| Acute non-ST segment elevation myocardial infarction \| \| Coronary Heart Disease \| G308 \| Inferior myocardial infarction NOS \| \| Coronary Heart Disease \| G309 \| Acute Q-wave infarct \| \| Coronary Heart Disease \| G30A \| Mural thrombosis \| \| Coronary Heart Disease \| G30B \| Acute posterolateral myocardial infarction \| \| Coronary Heart Disease \| G30X \| Acute transmural myocardial infarction of unspecif site \| \| Coronary Heart Disease \| G30X0 \| Acute ST segment elevation myocardial infarction \| \| Coronary Heart Disease \| G30y \| Other acute myocardial infarction \| \| Coronary Heart Disease \| G30y0 \| Acute atrial infarction \| \| Coronary Heart Disease \| G30y1 \| Acute papillary muscle infarction \| \| Coronary Heart Disease \| G30y2 \| Acute septal infarction \| \| Coronary Heart Disease \| G30yz \| Other acute myocardial infarction NOS \| \| Coronary Heart Disease \| G30z \| Acute myocardial infarction NOS \| \| Coronary Heart Disease \| G31 \| Other acute and subacute ischaemic heart disease \| \| Coronary Heart Disease \| G31-99 \| Acute/subacute IHD NOS \| \| Coronary Heart Disease \| G310 \| Postmyocardial infarction syndrome \| \| Coronary Heart Disease \| G310-1 \| Dressler's syndrome \| \| Coronary Heart Disease \| G311 \| Preinfarction syndrome \| \| Coronary Heart Disease \| G311-1 \| Crescendo angina \| \| Coronary Heart Disease \| G311-2 \| Impending infarction \| \| Coronary Heart Disease \| G311-3 \| Unstable angina \| \| Coronary Heart Disease \| G311-4 \| Angina at rest \| \| Coronary Heart Disease \| G3110 \| Myocardial infarction aborted \| \| Coronary Heart Disease \| G3110-1 \| MI - myocardial infarction aborted \| \| Coronary Heart Disease \| G3111 \| Unstable angina \| \| Coronary Heart Disease \| G3112 \| Angina at rest \| \| Coronary Heart Disease \| G3113 \| Refractory angina \| \| Coronary Heart Disease \| G3114 \| Worsening angina \| \| Coronary Heart Disease \| G3115 \| Acute coronary syndrome \| \| Coronary Heart Disease \| G311z \| Preinfarction syndrome NOS \| \| Coronary Heart Disease \| G312 \| Coronary thrombosis not resulting in myocardial infarction \| \| Coronary Heart Disease \| G31y \| Other acute and subacute ischaemic heart disease \| \| Coronary Heart Disease \| G31y0 \| Acute coronary insufficiency \| \| Coronary Heart Disease \| G31y0-99 \| Acute coronary syndrome \| \| Coronary Heart Disease \| G31y1 \| Microinfarction of heart \| \| Coronary Heart Disease \| G31y2 \| Subendocardial ischaemia \| \| Coronary Heart Disease \| G31y3 \| Transient myocardial ischaemia \| \| Coronary Heart Disease \| G31yz \| Other acute and subacute ischaemic heart disease NOS \| \| Coronary Heart Disease \| G32 \| Old myocardial infarction \| \| Coronary Heart Disease \| G32-1 \| Healed myocardial infarction \| \| Coronary Heart Disease \| G32-2 \| Personal history of myocardial infarction \| \| Coronary Heart Disease \| G33 \| Angina pectoris \| \| Coronary Heart Disease \| G330 \| Angina decubitus \| \| Coronary Heart Disease \| G3300 \| Nocturnal angina \| \| Coronary Heart Disease \| G330z \| Angina decubitus NOS \| \| Coronary Heart Disease \| G331 \| Prinzmetal's angina \| \| Coronary Heart Disease \| G331-1 \| Variant angina pectoris \| \| Coronary Heart Disease \| G332 \| Coronary artery spasm \| \| Coronary Heart Disease \| G33z \| Angina pectoris NOS \| \| Coronary Heart Disease \| G33z0 \| Status anginosus \| \| Coronary Heart Disease \| G33z1 \| Stenocardia \| \| Coronary Heart Disease \| G33z2 \| Syncope anginosa \| \| Coronary Heart Disease \| G33z3 \| Angina on effort \| \| Coronary Heart Disease \| G33z4 \| Ischaemic chest pain \| \| Coronary Heart Disease \| G33z5 \| Post infarct angina \| \| Coronary Heart Disease \| G33z6 \| New onset angina \| \| Coronary Heart Disease \| G33z7 \| Stable angina \| \| Coronary Heart Disease \| G33zz \| Angina pectoris NOS \| \| Coronary Heart Disease \| G34 \| Other chronic ischaemic heart disease \| \| Coronary Heart Disease \| G34-99 \| Chr. ischaemic heart dis. NOS \| \| Coronary Heart Disease \| G340 \| Coronary atherosclerosis \| \| Coronary Heart Disease \| G340-1 \| Triple vessel disease of the heart \| \| Coronary Heart Disease \| G340-2 \| Coronary artery disease \| \| Coronary Heart Disease \| G3400 \| Single coronary vessel disease \| \| Coronary Heart Disease \| G3401 \| Double coronary vessel disease \| \| Coronary Heart Disease \| G342 \| Atherosclerotic cardiovascular disease \| \| Coronary Heart Disease \| G343 \| Ischaemic cardiomyopathy \| \| Coronary Heart Disease \| G344 \| Silent myocardial ischaemia \| \| Coronary Heart Disease \| G34y \| Other specified chronic ischaemic heart disease \| \| Coronary Heart Disease \| G34y0 \| Chronic coronary insufficiency \| \| Coronary Heart Disease \| G34y1 \| Chronic myocardial ischaemia \| \| Coronary Heart Disease \| G34yz \| Other specified chronic ischaemic heart disease NOS \| \| Coronary Heart Disease \| G34z \| Other chronic ischaemic heart disease NOS \| \| Coronary Heart Disease \| G34z0 \| Asymptomatic coronary heart disease \| \| Coronary Heart Disease \| G35 \| Subsequent myocardial infarction \| \| Coronary Heart Disease \| G350 \| Subsequent myocardial infarction of anterior wall \| \| Coronary Heart Disease \| G351 \| Subsequent myocardial infarction of inferior wall \| \| Coronary Heart Disease \| G353 \| Subsequent myocardial infarction of other sites \| \| Coronary Heart Disease \| G35X \| Subsequent myocardial infarction of unspecified site \| \| Coronary Heart Disease \| G36 \| Certain current complication follow acute myocardial infarct \| \| Coronary Heart Disease \| G360 \| Haemopericardium/current comp folow acut myocard infarct \| \| Coronary Heart Disease \| G361 \| Atrial septal defect/curr comp folow acut myocardal infarct \| \| Coronary Heart Disease \| G362 \| Ventric septal defect/curr comp fol acut myocardal infarctn \| \| Coronary Heart Disease \| G363 \| Ruptur cardiac wall w'out haemopericard/cur comp fol ac MI \| \| Coronary Heart Disease \| G364 \| Ruptur chordae tendinae/curr comp fol acute myocard infarct \| \| Coronary Heart Disease \| G365 \| Rupture papillary muscle/curr comp fol acute myocard infarct \| \| Coronary Heart Disease \| G366 \| Thrombosis atrium,auric append&vent/curr comp foll acute MI \| \| Coronary Heart Disease \| G38 \| Postoperative myocardial infarction \| \| Coronary Heart Disease \| G380 \| Postoperative transmural myocardial infarction anterior wall \| \| Coronary Heart Disease \| G381 \| Postoperative transmural myocardial infarction inferior wall \| \| Coronary Heart Disease \| G382 \| Postoperative transmural myocardial infarction other sites \| \| Coronary Heart Disease \| G383 \| Postoperative transmural myocardial infarction unspec site \| \| Coronary Heart Disease \| G384 \| Postoperative subendocardial myocardial infarction \| \| Coronary Heart Disease \| G38z \| Postoperative myocardial infarction, unspecified \| \| Coronary Heart Disease \| G3y \| Other specified ischaemic heart disease \| \| Coronary Heart Disease \| G3z \| Ischaemic heart disease NOS \| \| Coronary Heart Disease \| G501 \| Post infarction pericarditis \| \| Coronary Heart Disease \| Gyu34 \| [X]Acute transmural myocardial infarction of unspecif site \| \| Stroke or TIA \| F4236 \| Amaurosis fugax \| \| Stroke or TIA \| Fyu55 \| [X]Other transnt cerebral ischaemic attacks+related syndroms \| \| Stroke or TIA \| G63y0 \| Cerebral infarct due to thrombosis of precerebral arteries \| \| Stroke or TIA \| G63y1 \| Cerebral infarction due to embolism of precerebral arteries \| \| Stroke or TIA \| G64 \| Cerebral arterial occlusion \| \| Stroke or TIA \| G64-1 \| CVA - cerebral artery occlusion \| \| Stroke or TIA \| G64-2 \| Infarction - cerebral \| \| Stroke or TIA \| G64-3 \| Stroke due to cerebral arterial occlusion \| \| Stroke or TIA \| G640 \| Cerebral thrombosis \| \| Stroke or TIA \| G6400 \| Cerebral infarction due to thrombosis of cerebral arteries \| \| Stroke or TIA \| G641 \| Cerebral embolism \| \| Stroke or TIA \| G641-1 \| Cerebral embolus \| \| Stroke or TIA \| G6410 \| Cerebral infarction due to embolism of cerebral arteries \| \| Stroke or TIA \| G64z \| Cerebral infarction NOS \| \| Stroke or TIA \| G64z-1 \| Brainstem infarction NOS \| \| Stroke or TIA \| G64z-2 \| Cerebellar infarction \| \| Stroke or TIA \| G64z-99 \| Cerebral A. occlusion NOS \| \| Stroke or TIA \| G64z0 \| Brainstem infarction \| \| Stroke or TIA \| G64z1 \| Wallenberg syndrome \| \| Stroke or TIA \| G64z1-1 \| Lateral medullary syndrome \| \| Stroke or TIA \| G64z2 \| Left sided cerebral infarction \| \| Stroke or TIA \| G64z3 \| Right sided cerebral infarction \| \| Stroke or TIA \| G64z4 \| Infarction of basal ganglia \| \| Stroke or TIA \| G65 \| Transient cerebral ischaemia \| \| Stroke or TIA \| G65-1 \| Drop attack \| \| Stroke or TIA \| G65-2 \| Transient ischaemic attack \| \| Stroke or TIA \| G65-3 \| Vertebro-basilar insufficiency \| \| Stroke or TIA \| G65-99 \| Transient Ischaemic Attacks \| \| Stroke or TIA \| G650 \| Basilar artery syndrome \| \| Stroke or TIA \| G650-1 \| Insufficiency - basilar artery \| \| Stroke or TIA \| G652 \| Subclavian steal syndrome \| \| Stroke or TIA \| G653 \| Carotid artery syndrome hemispheric \| \| Stroke or TIA \| G654 \| Multiple and bilateral precerebral artery syndromes \| \| Stroke or TIA \| G656 \| Vertebrobasilar insufficiency \| \| Stroke or TIA \| G65y \| Other transient cerebral ischaemia \| \| Stroke or TIA \| G65z \| Transient cerebral ischaemia NOS \| \| Stroke or TIA \| G65z-99 \| Transient Ischaemic Attacks \| \| Stroke or TIA \| G65z0 \| Impending cerebral ischaemia \| \| Stroke or TIA \| G65z1 \| Intermittent cerebral ischaemia \| \| Stroke or TIA \| G65zz \| Transient cerebral ischaemia NOS \| \| Stroke or TIA \| G66 \| Stroke and cerebrovascular accident unspecified \| \| Stroke or TIA \| G66-1 \| CVA unspecified \| \| Stroke or TIA \| G66-2 \| Stroke unspecified \| \| Stroke or TIA \| G66-3 \| CVA - Cerebrovascular accident unspecified \| \| Stroke or TIA \| G66-98 \| Stroke/CVA - undefined \| \| Stroke or TIA \| G66-99 \| Stroke \| \| Stroke or TIA \| G667 \| Left sided CVA \| \| Stroke or TIA \| G668 \| Right sided CVA \| \| Stroke or TIA \| G6760 \| Cereb infarct due cerebral venous thrombosis, nonpyogenic \| \| Stroke or TIA \| G6W \| Cereb infarct due unsp occlus/stenos precerebr arteries \| \| Stroke or TIA \| G6X \| Cerebrl infarctn due/unspcf occlusn or sten/cerebrl artrs \| \| Stroke or TIA \| Gyu63 \| [X]Cerebrl infarctn due/unspcf occlusn or sten/cerebrl artrs \| \| Stroke or TIA \| Gyu64 \| [X]Other cerebral infarction \| \| Stroke or TIA \| Gyu65 \| [X]Occlusion and stenosis of other precerebral arteries \| \| Stroke or TIA \| Gyu66 \| [X]Occlusion and stenosis of other cerebral arteries \| \| Stroke or TIA \| ZV12D \| [V]Personal history of transient ischaemic attack \| |
| --- | --- | --- | --- | --- | --- | --- | --- | --- | --- | --- | --- | --- | --- | --- | --- | --- | --- | --- | --- | --- | --- | --- | --- | --- | --- | --- | --- | --- | --- | --- | --- | --- | --- | --- | --- | --- | --- | --- | --- | --- | --- | --- | --- | --- | --- | --- | --- | --- | --- | --- | --- | --- | --- | --- | --- | --- | --- | --- | --- | --- | --- | --- | --- | --- | --- | --- | --- | --- | --- | --- | --- | --- | --- | --- | --- | --- | --- | --- | --- | --- | --- | --- | --- | --- | --- | --- | --- | --- | --- | --- | --- | --- | --- | --- | --- | --- | --- | --- | --- | --- | --- | --- | --- | --- | --- | --- | --- | --- | --- | --- | --- | --- | --- | --- | --- | --- | --- | --- | --- | --- | --- | --- | --- | --- | --- | --- | --- | --- | --- | --- | --- | --- | --- | --- | --- | --- | --- | --- | --- | --- | --- | --- | --- | --- | --- | --- | --- | --- | --- | --- | --- | --- | --- | --- | --- | --- | --- | --- | --- | --- | --- | --- | --- | --- | --- | --- | --- | --- | --- | --- | --- | --- | --- | --- | --- | --- | --- | --- | --- | --- | --- | --- | --- | --- | --- | --- | --- | --- | --- | --- | --- | --- | --- | --- | --- | --- | --- | --- | --- | --- | --- | --- | --- | --- | --- | --- | --- | --- | --- | --- | --- | --- | --- | --- | --- | --- | --- | --- | --- | --- | --- | --- | --- | --- | --- | --- | --- | --- | --- | --- | --- | --- | --- | --- | --- | --- | --- | --- | --- | --- | --- | --- | --- | --- | --- | --- | --- | --- | --- | --- | --- | --- | --- | --- | --- | --- | --- | --- | --- | --- | --- | --- | --- | --- | --- | --- | --- | --- | --- | --- | --- | --- | --- | --- | --- | --- | --- | --- | --- | --- | --- | --- | --- | --- | --- | --- | --- | --- | --- | --- | --- | --- | --- | --- | --- | --- | --- | --- | --- | --- | --- | --- | --- | --- | --- | --- | --- | --- | --- | --- | --- | --- | --- | --- | --- | --- | --- | --- | --- | --- | --- | --- | --- | --- | --- | --- | --- | --- | --- | --- | --- | --- | --- | --- | --- | --- | --- | --- | --- | --- | --- | --- | --- | --- | --- | --- | --- | --- | --- | --- | --- | --- | --- | --- | --- | --- | --- | --- | --- | --- | --- | --- | --- | --- | --- | --- | --- | --- | --- | --- | --- | --- | --- | --- | --- | --- | --- | --- | --- | --- | --- | --- | --- | --- | --- | --- | --- | --- | --- | --- | --- | --- | --- | --- | --- | --- | --- | --- | --- | --- | --- | --- | --- | --- | --- | --- | --- | --- | --- | --- | --- | --- | --- | --- | --- | --- | --- | --- | --- | --- | --- | --- | --- | --- | --- | --- | --- | --- | --- | --- | --- | --- | --- | --- | --- | --- | --- | --- | --- | --- | --- | --- | --- | --- | --- | --- | --- | --- | --- | --- | --- | --- | --- | --- | --- | --- | --- | --- | --- | --- | --- | --- | --- | --- | --- | --- | --- | --- | --- | --- | --- | --- | --- | --- | --- | --- | --- | --- | --- | --- | --- | --- | --- | --- | --- | --- | --- | --- | --- | --- | --- | --- | --- | --- | --- | --- | --- | --- | --- | --- | --- | --- | --- | --- | --- | --- | --- | --- | --- | --- | --- | --- | --- | --- | --- | --- | --- | --- | --- | --- | --- | --- | --- | --- | --- | --- | --- | --- | --- | --- | --- | --- | --- | --- | --- | --- | --- | --- | --- | --- | --- | --- | --- |
